# Catastrophic Brain Aspergillosis after Liver Transplantation

**DOI:** 10.1155/2021/8626057

**Published:** 2021-02-13

**Authors:** Mohammadmahdi Abbasi, Shirin Shams Ardakani, Nasir Fakhar, Hamid Eshaghi, Hosein Alimadadi, Toktam Faghihi, Behdad Gharib, Meisam Sharifzadeh, Masoomeh Safaei

**Affiliations:** ^1^Department of Pediatrics, Children‘s Mediacal Center, Tehran University of medical Sciences, Teran, Iran; ^2^Division of Liver Transplantation, Imam Khomeini Hospital, TUMS, Teran, Iran; ^3^Department of Pediatric Infectious Disease, Children‘s Medical Center, TUMS, Teran, Iran; ^4^Department of Pediatrics, Tehran University of medical Sciences, Teran, Iran; ^5^Children's Medical Center, Pediatrics Center of Excellence, Teran, Iran; ^6^Pediatric gastroenterology Research Center, Tehran University of medical Sciences, Teran, Iran; ^7^Department of Clinical Pharmacy, Children s Medical Center, TUMS, Teran, Iran; ^8^Division of Pediatric Critical Care, Children‘s Medical Center, TUMS, Teran, Iran; ^9^Pediatrician, Assistant professor of pediatric Intensive care, Division of pediatric critical care, Children medical center, Tehran University of medical science, Iran; ^10^Department of pathology, Cancer Institute, Imam Khomeini Hospital Complex, Tehran university of Medical Siences, Teran, Iran

## Abstract

*Background*. Liver transplantation has many complications. Because of receiving immunosuppressive regimens, infectious complications in these patients may have fatal results. Aspergillosis in solid organ recipients is one of the most common fungal infections that usually occur 1 month after transplantation. Aspergillus infection mainly involves the lungs. Although the central nervous system may get involved due to hematogenous spreading from lungs, isolated central nervous system involvement is rarely reported. *Case Presentation*. The patient was an 8-year-old boy, with a case of Wilson disease, who underwent liver transplantation due to acute fulminant hepatic failure. Four days after the surgery, he was affected by fever, agitation, loss of consciousness, hemiparesis, and focal seizure. Brain MRI showed abscess formation, whereas chest X-ray was normal. Intravenous antibiotics were initiated but the patient's condition was not improving; therefore, surgical drainage of the abscess was performed. The pathological investigation was compatible with aspergillosis. Antifungal therapy with voriconazole was administrated. His symptoms were resolved but unfortunately, brain lesions caused persistent vegetative state. *Discussion*. Aspergillus is a ubiquitous organism that mainly occurs in immunocompromised patients. Aspergillosis could be prevented by environmental modification such as installing high-efficiency particulate air filters. Chemoprevention with triazoles, echinocandins, and polyenes is also effective. Voriconazole is the drug of choice for aspergillosis treatment. Although voriconazole is a highly effective antifungal drug, cerebral aspergillosis is often fatal.

## 1. Background

Liver transplantation has become a more successful way to manage end-stage liver disease over the past few decades. Therefore, the importance of being aware of its complications is an even greater concern for physicians all over the world. Liver transplantation for end-stage liver diseases in children is done from both living and cadaveric donors, and the rate of transplantation from living donors is higher in children as its complications. Candidates for liver transplantation are divided into 5 groups:

1. Extrahepatic cholestasis
2. Intrahepatic cholestasis
3. Metabolic diseases
4. Acute liver failure
5. *Others*: primary liver tumor and cystic fibrosis

The complications of liver transplantation include vascular complication, hepatic artery stenosis, hepatic artery thrombosis, portal vein thrombosis, portal vein stenosis, IVC stenosis, IVC thrombosis, biliary complication, biliary obstruction, bile leak, bile duct stones, mucocele, organ rejection, localized fluid collection, posttransplantation lymphoproliferative disorders, and infectious complications.

The immunosuppressive therapies for pediatric transplantation include administration of corticosteroids, calcineurin inhibitors (cyclosporine and tacrolimus), mycophenolate mofetil, sirolimus, and IL2 receptor inhibitors [1, 2]. Infectious complication after transplantation surgery is one of the most frequent causes of total morbidity and mortality. Between all infections, 50-60% are bacterial, 20-40% are viral, 5-15% are fungal, and less than 10% are due to parasites [3–5]. Mortality rates of invasive fungal infection (IFI) have ranged between 25% and 69% in orthotopic liver transplantation recipients [3, 6–19]. Candida species are responsible for about 80% of all invasive fungal infections [3, 9–16]. Aspergillus species cause about 10% to 20% of all invasive fungal infections [4, 13–16, 20–24]. The reported incidence of aspergillosis after OLT varies between 1.5% and 4% in most studies [3, 19, 22]. Clinical manifestations of aspergillosis include invasive pulmonary aspergillosis, invasive aspergillus sinusitis, cutaneous aspergillosis, and cerebral aspergillosis [3–5, 7, 13, 16, 20–22]. Although CNS involvement is a common situation due to disseminated invasive aspergillosis, isolated CNS involvement is rarely reported.

## 2. Case Presentation

**Figure 1:**
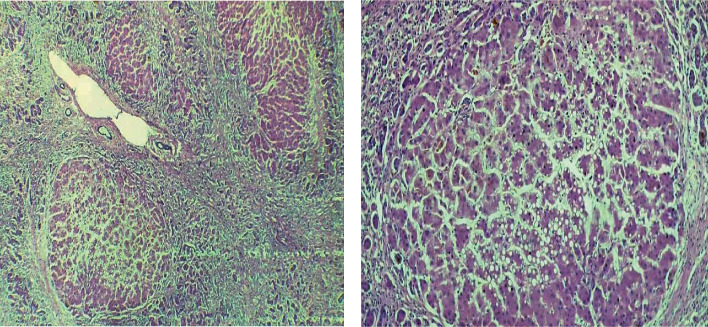
Microscopic examination shows liver tissue with diffuse nodular architecture, regenerative changes, marked ductular proliferation, and mild macrovesicular steatosis involves about 10 percent of liver tissue and scattered bile plugs (deposition of copper was not seen in our specimen).

**Figure 2:**
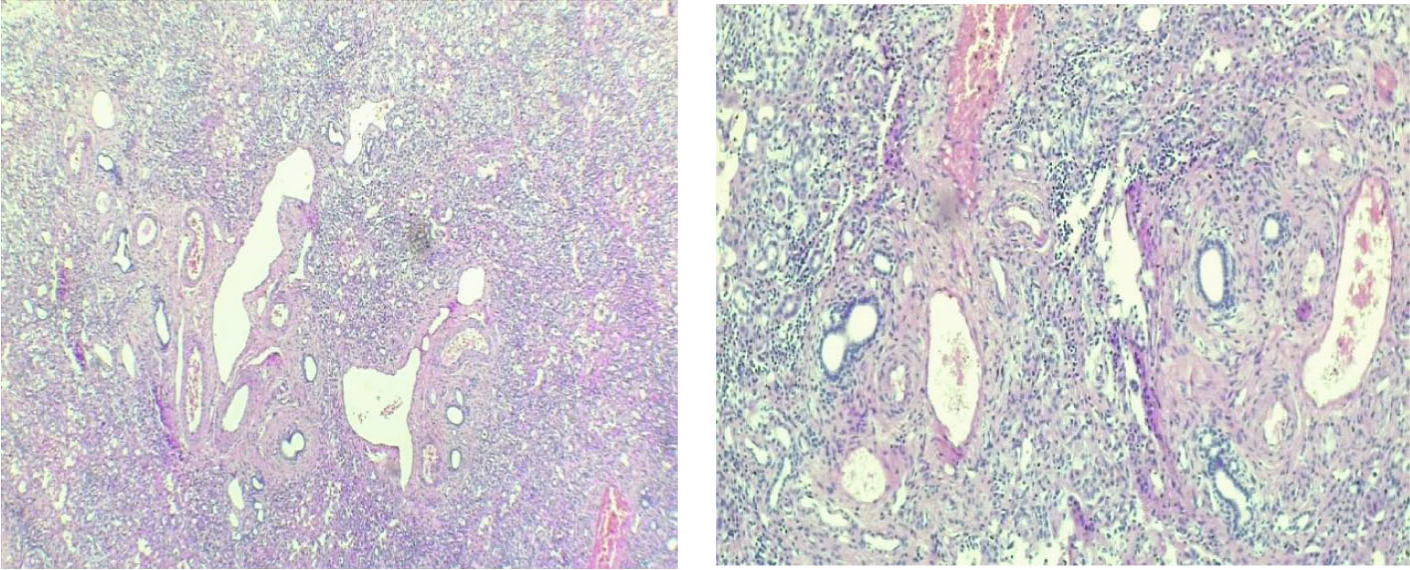
Interlobular bile ducts are preserved. Foci of hepatocyte confluent necrosis are present.

**Figure 3:**
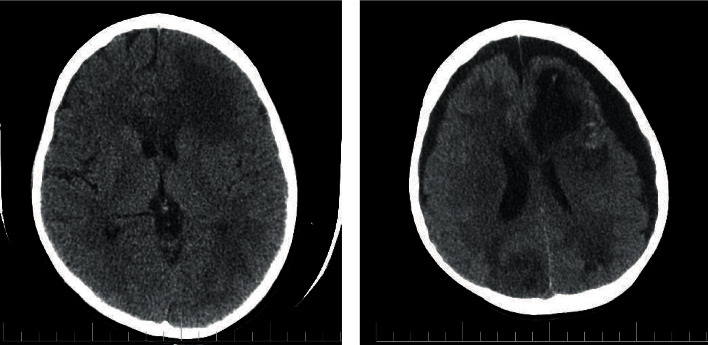
Brain CT scan: diffuse parenchymal hypodensity in left frontal and parietal lobes which suspected to a left-sided arterial stroke.

**Figure 4:**
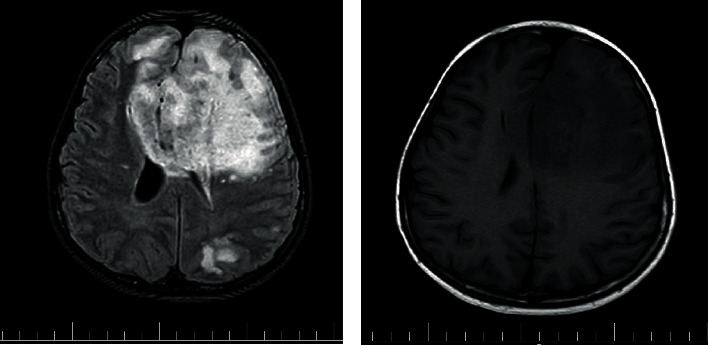
Brain MRI: diffuse abnormal signal foci had seen in both hemispheres, and the largest one is in the left frontal, left temporoparietal, and right occipital regions along with peripheral vasogenic edema.

The patient was an 8-year-old boy, with a case of Wilson disease proved by liver biopsy (Figures 1 and 2), who underwent liver transplantation from a deceased donor. Pretransplant screenings for donor and recipient such as HIV, HBV, HCV, HDV, CMV, EBV, and syphilis for donor and HIV, HAV, HBV, HCV, HDV, CMV, EBV, VZV, PPD, IGRA, and syphilis for the recipient were negative. After the surgery, the patient was admitted in ICU, and methylprednisolone, tacrolimus, piperacillin/tazobactam, vancomycin, ganciclovir, trimethoprim/sulfamethoxazole, and fluconazole were initiated. His vital signs were stable in ICU. His level of consciousness improved gradually, and after 72 hours, he was fully conscious; therefore, he had transferred to the posttransplant ward which some part of it was under construction without proper precautions. On postoperative day 4, his body temperature raised to 38.1°C. In addition to the fever, he presented agitation, disorientation, and loss of consciousness. Afterward, he experienced a right-sided hemiparesis and an episode of seizure presented with lateral gaze with tonic phase in upper and both of the lower limbs. He had a normal flexor plantar response. PRES (posterior reversible encephalopathy syndrome), brain ischemia, infectious diseases, and tacrolimus toxicity were suspected, so tacrolimus was discontinued and prednisolone and mycophenolate mofetil were initiated. His brain CT scan showed diffuse parenchymal hypodensity in left frontal and parietal lobes which suspected to a left-sided arterial stroke (Figure 3). Electroencephalography (EEG) showed multifocal sharp waves compatible with diffuse encephalopathy accompanied by predominantly right-sided hemiparesis. To get more information, brain MRI had done. Diffuse abnormal signal foci had seen in both hemispheres, and the largest one is in the left frontal, left temporoparietal, and right occipital regions along with peripheral vasogenic edema (Figure 4). Other small lesions are well-defined, round foci in white matter/gray matter (WM/GM) boundary which show T2 hyposignal rim. Brain MRI revealed multiple brain abscess formation. There might be a component of ischemic insult in the larger lesion with cortical and corpus callosum involvement. Laboratory tests were performed to rule out common infections after liver transplantation (Tables 1 and 2). In further evaluations, the patient's level of consciousness did not improve; therefore, he was transferred to ICU and underwent mechanical ventilation.

His level of electrolytes was balanced. Three days after transferring to ICU, due to prolonged fever and no response to antibiotic therapy, liposomal amphotericin B was added to his medication regimen. There was no proper clinical response to antimicrobial agents after 5 days of pharmacotherapy, so surgical drainage of brain lesions was performed. Abscess culture was positive for aspergillosis, and voriconazole was initiated, respectively. After about 20 days of ICU admission and antifungal agent administration, patient's brain function improved neither clinically nor radiologically. He did not have a fever any longer. The patient was successfully weaned from mechanical ventilation on the first attempt. His seizures had controlled. Unfortunately, the fungal lesion in his brain caused persistent vegetative state (PVS) and disseminated spasticity.

## 3. Discussion

Aspergillus is an ubiquitous organism that involves various organs in the human body. Invasive aspergillosis involves lungs (56% of symptomatic cases), skin (5% of symptomatic cases), paranasal sinuses (5% of symptomatic cases), and central nervous system (6% of symptomatic cases). Invasive aspergillosis affects immunocompromised patients mostly (0.4% of pediatric admissions) [25]. The risk of invasive aspergillosis in patients with malignancies (74%), hematologic diseases (28%), and immunologic diseases (18%) is higher than similar immunosuppressive conditions such as the patients who had undergone bone marrow transplantation (15%) and solid organ transplantation (1%) [25]. Considering the presence of the organism in external auditory canals or respiratory tract, isolated CNS aspergillosis could cause due to direct invasion of the CNS or hematogenous spreading [26]. Aspergillus is an ubiquitous organism that could be found in the environment; therefore, one of the most important solutions to prevent invasive aspergillosis is controlling environmental factors. Installing high-efficiency particulate air filters (HEPA) makes a significant improvement in controlling nosocomial affliction [27, 28]. Triazoles (itraconazole, posaconazole, and voriconazole), echinocandins (caspofungin, micafungin, and anidulafungin), and polyenes (amphotericin B deoxycholate and liposomal amphotericin B) are used to prevent invasive aspergillosis (IA) as chemoprevention regimen. Although these prophylactic drugs have reduced invasive fungal infections, using them may cause other side effects, for example, using voriconazole has been associated with increasing zygomycosis in several studies [29–31]. Schwartz and colleagues treated patients with definite or possible central nervous system (CNS) aspergillosis with voriconazole which 35% of the patients responded completely or partially [32]. According to the study of Walsh and colleagues, voriconazole is recommended as first-line treatment and other antifungal agents such as other triazoles will be used in case the patient is intolerant or refractory to voriconazole [33]. Except for visual disturbances and skin reactions, other systemic side effects are more frequent in patients who received amphotericin B [31, 34]. CNS aspergillosis in this patient was treated by voriconazole, and surgical drainage also had done. Unfortunately, the brain lesion had destructive effects on the cerebral cortex and caused loss of consciousness and other neurological defects.

## Figures and Tables

**Table 1:** Postoperation flow cytometry.

CD10	Negative	CD20	6 (%)	CD64	5 (%)
CD117	Negative	CD22	5 (%)	CD7	11 (%)
CD11b	80 (%)	CD23 5a	Negative	CD71	3 (%)
CD13	55 (%)	CD3	11 (%)	CD8	4 (%)
CD14	3 (%)	CD33	8 (%)	HLA-DR	11 (%)
CD15	78 (%)	CD34	Negative	MPO	79 (%)
CD19	6 (%)	CD4	6 (%)	TdT	Negative
CD2	11 (%)	CD5	10 (%)	Cyto CD79a	9 (%)

**Table 2:** Intensive care unit laboratory data.

1^st^ day	White cell count	24.69 (×10^3^/*μ*l)	C reactive protein (quantitative)	19 (mg/l)
	Hemoglobin	11.3 (gr/dl)	Erythrocyte sedimentation rate-1 hr	26 (mm/h)
	Platelet count	80 (×10^3^/*μ*l)	Blood culture	Negative after 24 hours
	Neutrophils	78.3 (%)	Lymphocytes	14.3 (%)

3^rd^ day	Cytomegalovirus R/T PCR	Undetectable	Blood culture	Candida SP.
	Interferon gamma release assay	Negative	Galactomannan	0.46

6^th^ day	Hydatic cyst IgM	Negative	Hydatic cyst IgG	Negative

8^th^ day	Toxoplasma IgM	0.1 (IU/ml)	Toxoplasma IgG	21.4 (IU/ml)

11^th^ day	Epstein barr virus R/T PCR	Undetectable	Pneumococci PCR	Undetectable
	Staphylococci PCR	Undetectable	Tuberculosis PCR	Undetectable
